# Supplementary figures and images for: Single-cell amplicon sequencing reveals community structures and transmission trends of protist-associated bacteria in a termite host
Source: PLoS One. 2020 May 15;15(5):e0233065. doi: 10.1371/journal.pone.0233065 (PMC7228121; doi:10.1371/journal.pone.0233065)

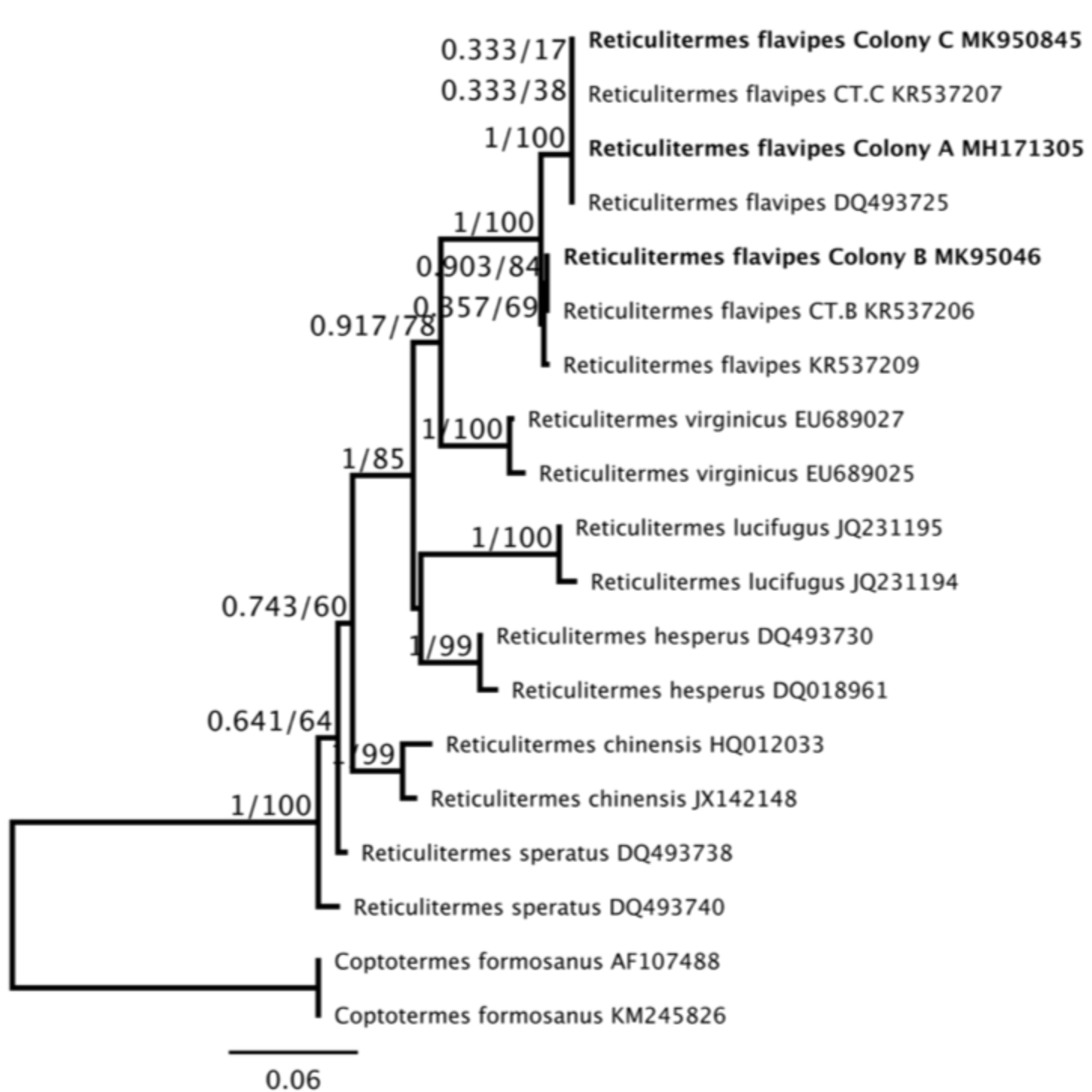

Supplement: S1 Fig — Sequences were aligned to references with MUSCLE and a Maximum likelihood (ML) phylogenetic tree was made using IQ-Tree with substitution model TIM2+G4. Sequences obtained from termites used in this study (Bold) clustered within the R. flavipes clade. Support values represent the Bayesian posterior probability and Bootstrap support values respectively. (TIF) [file pone.0233065.s003.tif]

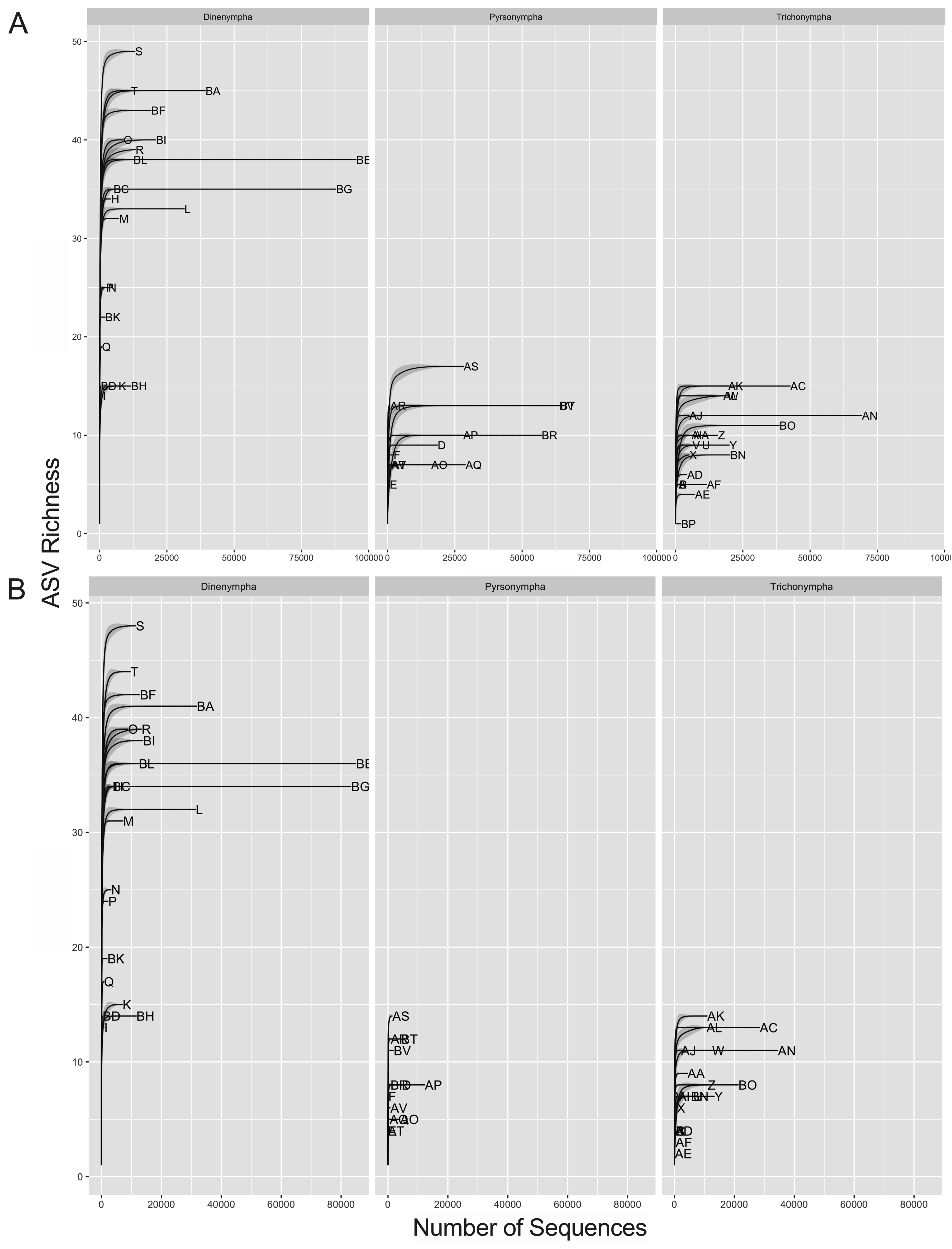

Supplement: S2 Fig — Analysis was done using the “ggrare” function of ggplot2 samples with (A) and without (B) sequences of ‘Candidatus Endomicrobium’. Curve tips represent the cell’s identification. (TIF) [file pone.0233065.s004.tif]

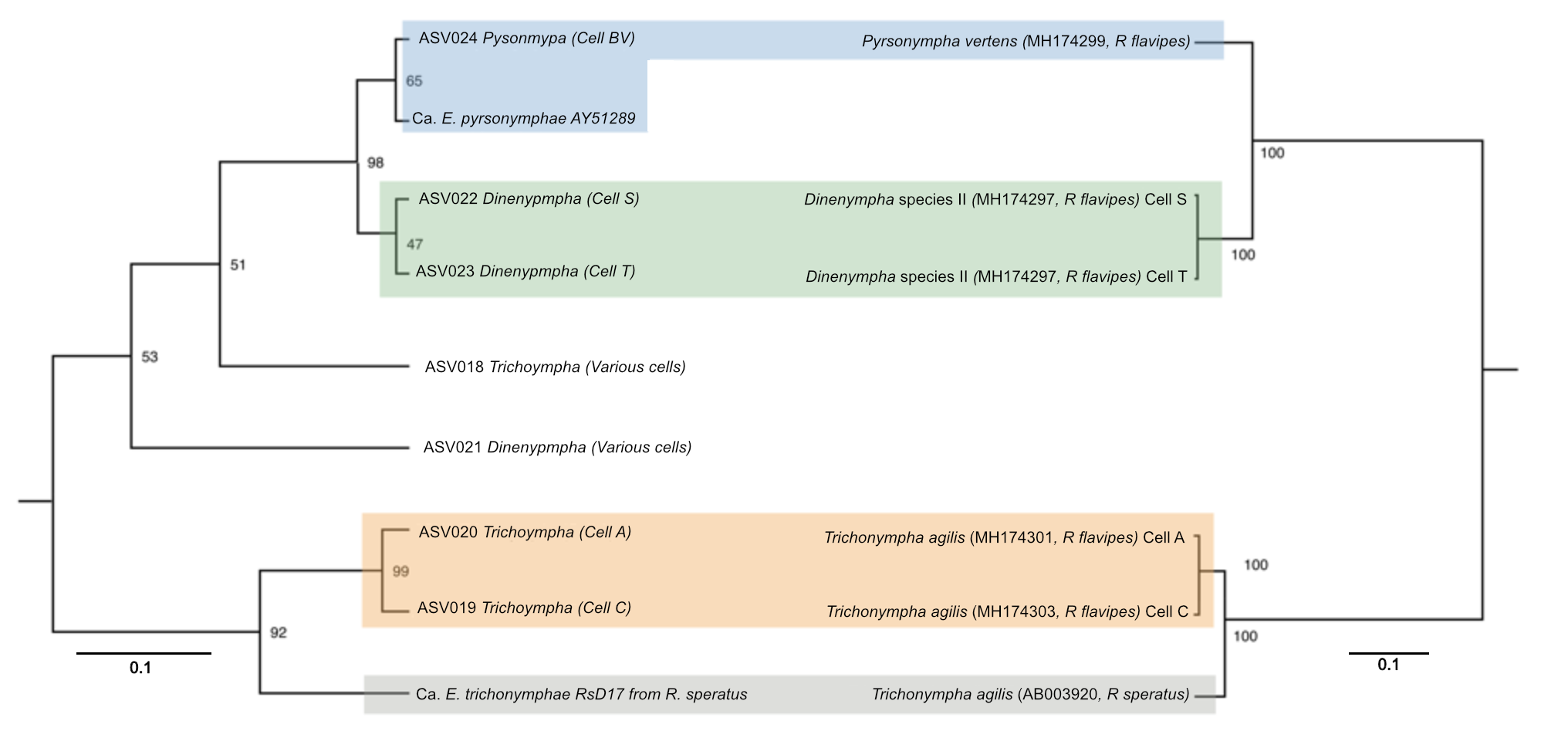

Supplement: S3 Fig — Endomicrobium ASVs were aligned to the V4 regions of reference Endomicrobium species (left tree). The phylogenetic tree was made using Jukes-Cantor Distance Model with UPGMA as implemented in Geneious v9. The 18S rRNA genes from single protist hosts, and one reference sequence were aligned, and the tree was made using Jukes-Cantor Distance Model with UPGMA as implemented in Geneious v9 (right tree). Support values are Bootstrap percentage values. Sequences are color coded such endosymbionts are colored the same as their hosts. In the left tree, Endomicrobium ASVs are given host protist designations, and a host cell identification is given. These host cells contained the ASV as the majority, or only, member of the Endomicrobium associated with that cell. Other host cells (not indicated here but detailed in S1 File) also contained the Endomicrobium ASVs mapped here as sole, or majority, members of the association. Sequences of the Endomicrobium ASVs can be found in S1 File. (TIF) [file pone.0233065.s005.tif]

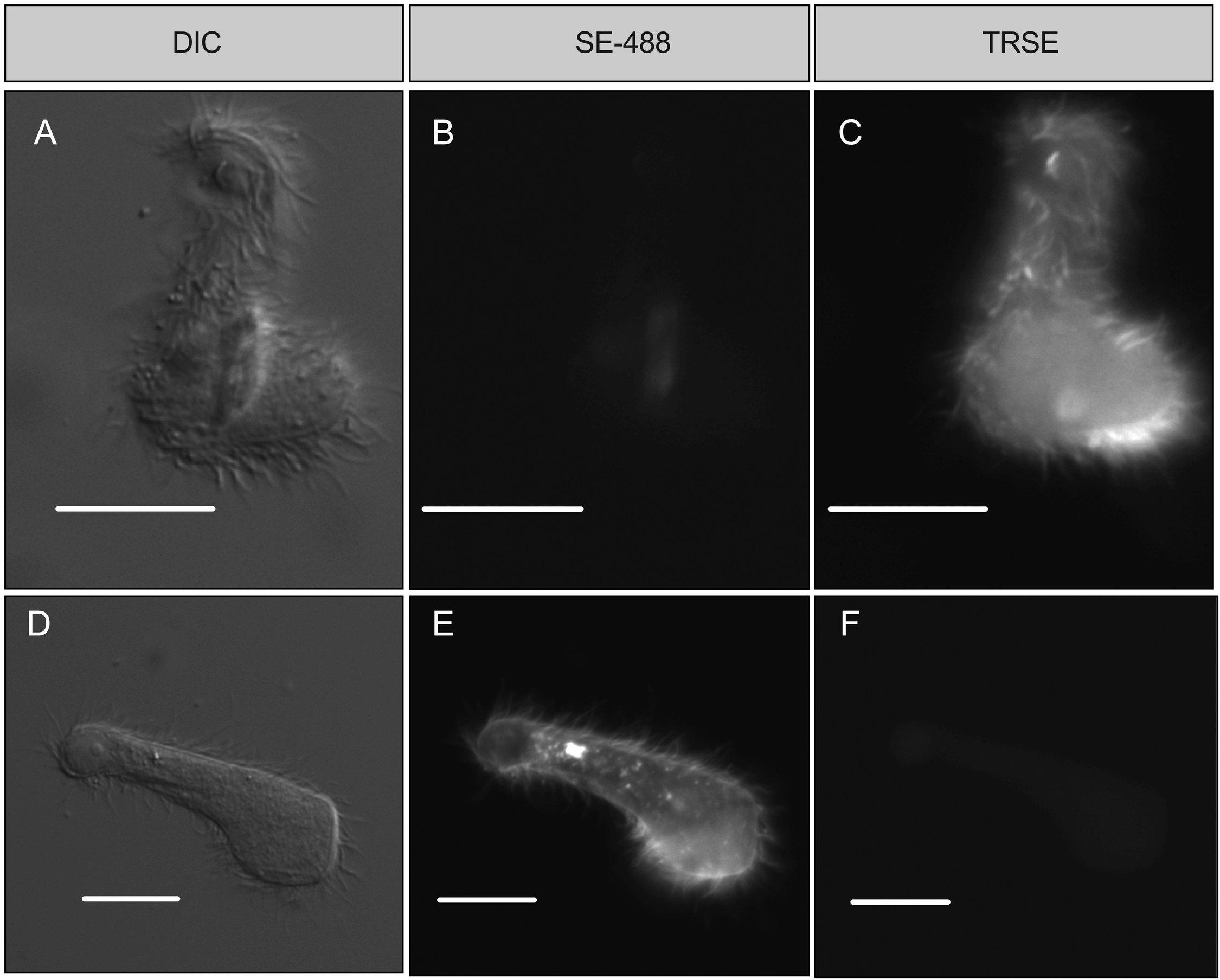

Supplement: S4 Fig — Representative micrographs of protist cells from non-mixed control samples from fluorescent assays. Micrographs represent DIC (A and D) SE-488 fluorescence (B and E) and TRSE fluorescence (C and D). Control samples always maintained homogenous fluorescence. Micrographs were taken post 12 hours from the start of assays and scale bars represent 20μm. (TIF) [file pone.0233065.s006.tif]
